# Expression of mRNA IL-17F and sIL-17F in atopic asthma patients

**DOI:** 10.1186/s13104-017-2517-9

**Published:** 2017-06-12

**Authors:** Mochammad Hatta, Eko E. Surachmanto, Andi Asadul Islam, Syarifuddin Wahid

**Affiliations:** 1 Molecular Biology and Immunology Laboratory, Faculty of Medicine, Hasanuddin University, Makassar, Indonesia; 2 Allergy Immunology Division, Department of Internal Medicine, Faculty of Medicine, Sam Ratulangi University, Manado, Indonesia; 3 Department of Neurosurgery, Faculty of Medicine, Hasanuddin University, Makassar, Indonesia; 4 Department of Pathology Anatomy, Faculty of Medicine, Hasanuddin University, Makassar, Indonesia

**Keywords:** mRNA IL-17F, Soluble IL-17F, Asthma, ELISA, Real-time PCR

## Abstract

**Background:**

Asthma is a chronic inflammatory disorder of airway that involves many cells and elements. Chronic inflammation caused by increase Airway *hyperresponsiveness* that cause recurrent episodic symptoms of breathlessness, wheezing, chest tightness and coughing, especially at night or early morning. Interleukin 17F is a cytokine that plays an important role in the pathophysiology of asthma attacks. Some studies show a variety of IL-17F roles in the pathogenesis of airway inflammation due to an allergic reaction.

**Results:**

The study was conducted at the Prof. Dr. R. D. Kandou Manado Hospital, Indonesia. Samples were taken continuously until the number of meant samples was achieved. Blood samples were collected from 40 atopic asthmatic patients. From statistical analysis based on the hypothesis, there was positive correlation between mRNA levels of IL-17F and IL-17F in atopic asthmatic patient (p = 0.000 and r = 0.988).

**Conclusions:**

According these data suggest that levels of mRNA IL-17F and IL17F might be useful parameters for the diagnosis of atopic asthma patient.

## Background

Asthma is a chronic inflammatory disorder of airways which involves many cells and elements. This chronic inflammation caused by increase airway hyperresponsiveness will manifest as recurrent episodic symptoms of breathlessness, chest tightness, wheezing and coughing, especially at night or early morning. Asthma is an important and serious public health problem in many countries in the world. Asthma can be mild and doesn’t interfere with the activity, but it can also be persistent and disturbing [1–3]. Atopic asthma or allergic asthma is asthma based on allergic reaction to the acquisition properties of atopy that found in family and characterized by increased total immunoglobulin E (IgE) or specific IgE. The prevalence of asthma in Indonesia ranged between 5 and 7%, in global prevalence of asthma ranging from 7 to 10%. According to data from the Center for Diseases Control and Prevention (CDC) National Asthma Control Program in United States 2010 there were 18.7 million adults suffer from Asthma and about nine people die from asthma each day [1, 2, 4, 5]. The prevalence of atopic asthma between 70 and 80% of people with asthma, and about 70–80% of people with asthma also accompanied with allergic rhinitis. The development of science and technology in the world is not entirely followed by the progress treatment for asthma, this has been shown by the data from many countries which show that number of asthma visit in emergency department, hospitalizations, morbidity and even mortality had increased [5]. In the developed country asthma exacerbations usually is caused by a viral infection, this is different from developing countries that exacerbations are usually caused by bacteria [1, 6].

Interleukin 17F is a cytokine that plays an important role in the pathophysiology of asthma attacks. Some studies has shown variety of IL-17F roles in the pathogenesis of airway inflammation due to an allergic reaction. Interleukin 17F plays role in asthma attacks through various channels, such as affect the levels of respiratory tract neutrophil, stimulates mucus hypersecretion, respiratory hyperreactivity, airway remodeling, and resistance from steroid [7–10]. Various gene variations associated with the occurrence of asthma and asthma severity, however the genetic role of mRNA IL-17F and IL-17F has not been fully understood [11]. This study shows that mRNA IL-17F levels and soluble IL-17F was higher in patients with asthma compared with the control group, although it has not been able to mention relationship with the severity of asthma.

## Materials and methods

### Subjects

The study was conducted in allergy immunology clinic at Prof. Dr. R. D. Kandou Manado Hospital. After receiving approval from the hospital institutional review board, we obtained forty patients who attending allergy immunology clinic from April to October 2015. The sample selection is done by purposive sampling studies and cross-sectional study design. Informed consent was obtained from the patients.

### Methods

The diagnosis of asthma is made by anamnesis history of asthma, physical examination and lung function measurements using peak expiratory flow meter based on criteria of GINA. Atopy Diagnosis is based on total serum IgE levels ≥100 IU/mL, measured using enzyme-linked immunosorbent assay (ELISA) or with a positive skin test to house dust ≥1/mites [12]. Skin testing is done by using a 1 ml syringe with a 26 G needle sizes that are injected on the volar forearm within 1.5–2.5 cm space between allergens and scored after 15–20 min. Positive values are obtained when the ratio of allergen extract of house dust with histamine as ≥0.5 from a positive control [13, 14]. Blood samples were drawn by vein puncture from the patients at least 5 ml of whole blood. Evaluation of serum interleukin 17F blood and mRNA IL 17F samples were taken from all participants and centrifuged at 400*g* for 10 min. Serum samples were stored at −70 °C and then the sample transferred to laboratory for analysis. Serum levels of soluble IL-17F measured using commercially available enzyme-linked immunosorbent assay kits (Catalog no. AB100557) according to the manufacturer’s instructions with detection range from 13.72 to 10.000 pg/ml [15]. Detection of mRNA gene IL-17F by using primary specific IL-17F forward: 5′-GTGCCAGGAGGTAGTATGAAGC-3′; IL-17F reverse: 5′-ATGTCTTCCTTCCTTG AGCATT-3′. PCR Protocol: by doubling DNA with 94 °C cycle in 3 min, repeated 38 times with 54 °C (30 s). Detection GAPDH gene by using primary forward/sense: 5′-AGTCAACGGATTTGGTCGTATT-3′; Protocol PCR: 94 °C (10 min); 32 cycle of 54 °C (30 s). Primary reverse/antisense; 5′-ATGGGTGGAATCATATTGGAAC-3′ according to Tomomi Yajima’s protocol. QRT-PCR is using the Green QRT-PCR master mix kit, one step. This protocol is optimized for the CFX Connect system, Biorad Laboratories instrument. The protocol is synced using instrument by changing coloring dilution according to the manual and following the producer company that is recommended for RT-PCR cycle program.

Passive coloring reference is put into the reaction, diluted 1:500. The solution, which contains coloring, was omitted from the light. Dilution 2x SYBR Green QRT-PCR master mix and stored on ice. Following early dilution master mix, the remaining unused part is stored in 4 °C with note, avoid solid–liquid cycle that is reduced. Reaction experiment is prepared by adding the following components. Preparing reagent mixture for reaction by using several components as follows. Reagent mixture by taking the last volume 25 μl (including RNA experiment) 12.5 μl from 2x SYBR Green QRT-PCR master mix added by 1 μl from early primary (optimized concentration) and also 0.375 μl of reference coloring mixture from the first step (optional) along with 1.0 μl from RT/Rnase mixture of block enzyme with 50 μl of total volume reaction can also be used. Reaction is mixed slowly to prevent any bubble formation (not rotated), and then mixture is distributed into reaction tubes by adding 1 μl RNA of experiment into each reaction tubes, reaction is mixed slowly to prevent any bubble formation (not rotated). Reaction is centrifuged shortly and placed into instrument and PCR program is ready to go by using Realtime PCR machine (CFX Connect system, Biorad Laboratories, Real Time PCR 96 well 0.1 ml, USA) [16, 17].

### Data analysis

Our study was designed to have 80% power with a type I error 5% to detect significant correlation between mRNA levels of IL-17F and IL-17F in atopic asthmatic patient Mean and standard deviations for normally distributed data were calculated. Correlations were performed using Pearson’s coefficient. All statistics were performed on IBM SPSS version 20 statistical software.

## Results

A total of 40 asthma patients have range of age: 18–60 years old (43.9 ± 11.03 years) were divided into 15 male and 25 female. Hemoglobin range level 12.4–17.9 mg % (14.3 ± 1.1). Neutrophil levels range level 29.4–71.80% (52.1 ± 9.75). Total IgE range level 144–6186% (1100.98 ± 1164). mRNA IL-I7F range level 9.30–21.22 (14.18 ± 3.35) copy/μ. IL-17F range level 71.40–1904 (916.94 ± 520) pg/ml (Table 1).

**Table 1:** Baseline characteristics of subject who suffer from atopic asthma

	Minimum	Maximum	Mean	Std. error	Std deviation
Age	23	60	43.90	1.745	11.038
Haemoglobin (mg%)	12.40	17.90	14.3500	0.18828	1.19078
Neutrophil	29.40	71.80	52.1555	1.54234	9.75458
IgE total	144	6186	1100.97	184.149	1164.662
IL-17F (pg/ml)	71.40	1904.03	916.9440	82.28120	520.39203
mRNA IL17F (pg/ml)	9.3	21.22	14.1822	53,072	3.35657

Among the subject in this study the mean level of IL-17F on male subjects is higher than female subjects 1044.09 vs 840.66 pg/ml. While the mean level of IL17 mRNA level was also higher among male subject.

### Correlation between serum mRNA IL-I7F with sIL-17F levels in atopic asthma patient

**Fig. 1:**
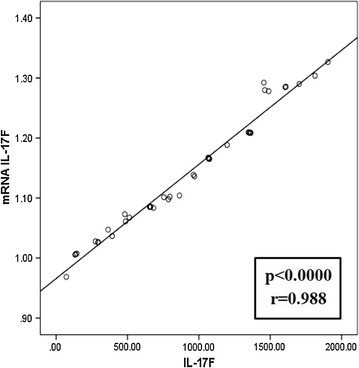
Scatter plot correlation between mRNA IL-17F levels and sIL-I7F in atopic asthma patient

Forty patients were enrolled with atopic asthma. All patient has sIL-17F range level of 71.40–1904 pg/ml (916.94 ± 520) and mRNA IL-I7F range level 9.30–21.22 copy/μ (14.18 ± 3.35). Because of data mRNA IL-17F and sIL-17F were normally distributed, to evaluate correlation between mRNA levels of sIL-17F and IL-17F Pearson correlation test was used. A p value of <0.05 was considered statistically significant. The results show that mRNA levels of IL-17F and sIL-17F were significantly up regulated in asthmatic patient (p < 0.000 and r 0.988) (Table 2). Scatter plot correlation between serum IL-17F with expression of mRNA IL-17F levels in atopic asthma F and patient is shown here (Fig. 1). These data suggest that the level of mRNA IL-17F and sIL-17F might be used as parameter for diagnosis of atopic asthma.

**Table 2:** The mean level of mRNA-IL17F and sIL-17F among male and female subjects who suffered from Atopy Asthma

	N	Mean mRNA-17F	Mean sIL-17F level
Male	15	14.95	1044.09
Female	25	13.72	840.66

## Discussion

Asthma is a disease which is usually diagnosed based on episodic symptoms and variable airway obstruction. It is also characterized by variable degrees of chronic inflammation and structural alterations in the airways [18–20]. This is a disease that usually affects kids and young adults at a higher frequency and is a cause of increased morbidity and mortality [21]. Asthma is no longer regarded as a pure inflammatory disease anymore rather it’s now regarded as a disease in which involved both inflammatory and structural changes [22]. These collective structural alterations known as airway remodeling, encompass various changes in lot of aspect of airway such as composition, content, and organization of many cellular and molecular constituents [23]. Airway remodeling encompasses lot of aspects such as epithelial detachment, goblet cell hyperplasia, subepithelial thickening, hyperplasia and hypertrophy of airway smooth muscle, bronchial gland enlargement, angiogenesis, and alterations in the extracellular matrix components. These changes is influenced by complicated network of cytokines [23, 24]. The clarification of the modulation of this cytokine network could contribute to the understanding of asthma pathogenesis and development of new therapeutic strategies. Asthma is a chronic inflammatory disorder of the airways which is heavily driven by T cell [9]. Both T helper (Th)2 and Th1 lymphocytes, play an important role in the pathophysiology of asthma.

The over production of T helper (Th)2 cytokines (IL-4, IL-5, IL-9 and IL-13) in the asthmatic airways is well defined, and recent studies indicate that IFN-γ which is produced by Th1 might responsible for severe airway inflammation. Recently, a separate T cell lineage, called Th17 cells or inflammatory T cells, producing IL-17A (or IL-17), has been identified. IL-17 is a 20 to 30-kD protein, the original member of the IL-17 cytokine family was first identified in 1993 and initially recognized for its similarity to a sequence belonging to the open reading frame 13 of *Herpes virus saimiri*. Five additional members, IL-17B, IL-17C, IL-17D, IL-17E (also called IL-25), and IL-17F, were discovered within a short period of time since 2000 to 2002. Th17 cell was thought to play an important role in the pathophysiology of atopic asthma [8, 10, 22]. IL-17F has an important role in the recruitment of neutrophils and in asthmatic patients, studies has shown that there is an increase of expression of IL-17F in the sputum, lung, bronchoalveolar lavage fluids and peripheral blood of asthmatic patients [17].

Previous study discovered that activated CD4 T cell, basophils and mast cell show increase in the expression of IL-17F. These three cell is an important regulatory cell types that play important role for regulating allergic airway inflammation in bronchial asthma. While IL-17F which is produced by Th17 cell constituting independent CD4^+^ T cell subset [18]. Among the IL-17 cytokine family members, IL-17F shows the highest amino acid sequence homology (50%) to IL-17A, while only 10–30% sequence identity is seen between IL-17A and the other family members [7]. The IL-17F gene was discovered in 2001, and located on chromosome 6p12. IL17A and IL17F gene was located in same chromosome region. However, IL 17F seems to function differently from IL-17A in immune response and disease [25]. IL-17F is derived from activated CD4+ cells, basophil and mast cell which are important regulatory cell types for allergic airway inflammation. Increased expression of IL-17F after segmental allergen challenge is seen in the bronchoalveolar lavage cells from asthmatic person. IL-17 F is expressed in both epithelium and inflammatory infiltrates [26, 27]. Immunocytochemistry showed that IL-17F positive cells in the subepithelial component and epithelium are significantly elevated in severe asthma compared with healthy and mild asthmatic subjects [27]. Additionally, an increased expression of epithelial IL-17F was correlated with disease severity. Another recent study demonstrated that asthmatic patients have a significantly higher level of serum IL-17 F protein as compared to that of healthy subjects [28]. This implies that IL-17F can be used as a clinical biomarker for asthma diagnosis and management. IL-17F was thought to have a role in the enhancement of allergic airway inflammation. This role was hypothesized due to finding that IL-17F has been shown to be able to induce pulmonary neutrophilia and produce an additive effect on antigen-induced allergic inflammatory response. IL-17F is also able to induce several cytokines and chemokines in bronchial epithelial cells, vein endothelial cells and fibroblast. IL-17F will affects the eosinophil, to induced several cytokines and chemokines such as IL-1β, IL-6, IL-8, GRO α, and MIP-1 β.

Another evidence for the role of sIL-17F in the pathogenesis of asthma is provided by the increase of IL-17mRNA in airways of mouse model of asthma and also in the sputum of asthmatic patients. Increase of IL-17mRNA is heavily correlated with number of neutrophils and consistent with its mRNA expression [29, 30]. Research demonstrated that in inflamed bronchi induced by allergen inhalation the IL-17F mRNA expression will be upregulated, and that sIL-17F differentially regulates bronchial influx of granulocytes induced by allergen both after single as well as after repeated allergen inhalation by sensitized mice. Rapid production of mRNA for IL-17F after allergen provocation suggested the presence of sIL-17F producing memory T cell population in inflamed airways.

## Conclusions

This study show that mRNA levels of IL-17F and sIL-17F has significantly correlation in atopic asthmatic patient (p = 0.000 and r = 0.988). These data suggest that the levels of mRNA IL17F and sIL17F might be useful parameters for the diagnosis of atopic asthma patient.

## Abbreviations

CD: cluster of differentiation
CDC: Center for Diseases Control and Prevention
ELISA: enzyme-linked immunosorbent assay
GINA: global initiative for asthma
IFN: interferon
Ig: immunoglobulin
IL: interleukin
mRNA: messenger RNA
PCR: polymerase chain reaction
RT: reverse transcriptase
sIL: soluble interleukin
Th: T helper
